# The Contemporary Role of Salvage Radical Prostatectomy in the Management of Recurrent Prostate Cancer: An Up-to-Date Review

**DOI:** 10.3390/life14070868

**Published:** 2024-07-11

**Authors:** Stamatios Katsimperis, Alexandros Pinitas, Maria Zerva, Themistoklis Bellos, Ioannis Manolitsis, Georgios Feretzakis, Vassilios S. Verykios, Ioannis Kyriazis, Panagiotis Neofytou, Sotirios Kapsalos, Panagiotis Deligiannis, Panagiotis Triantafyllou, Patrick Juliebø-Jones, Bhaskar Somani, Iraklis Mitsogiannis, Lazaros Tzelves

**Affiliations:** 1 Second Department of Urology, Sismanoglio Hospital, National and Kapodistrian University of Athens, 15126 Athens, Greece; bellos.themistoklis@gmail.com (T.B.); giannismanolit@gmail.com (I.M.); ioanniskyriazis93@gmail.com (I.K.); neo.panagiotis@gmail.com (P.N.); kapsalos13@gmail.com (S.K.); panosdlg@gmail.com (P.D.); panos30fyllu@gmail.com (P.T.); imitsog@med.uoa.gr (I.M.); lazarostzelves@gmail.com (L.T.); 2 Department of Urology, General Hospital of Athens “G. Gennimatas”, 11527 Athens, Greece; alexpinitas@gmail.com; 3 Department of Urology, Red Cross General Hospital of Athens, 11526 Athens, Greece; mariazegr@gmail.com; 4 School of Science and Technology, Hellenic Open University, 26335 Patras, Greece; georgios.feretzakis@ac.eap.gr (G.F.); verykios@eap.gr (V.S.V.); 5 Department of Urology, Haukeland University Hospital, 5009 Bergen, Norway; jonesurology@gmail.com; 6 Department of Urology, University Hospital, Southampton SO16 6YD, UK; bhaskarsomani@yahoo.com

**Keywords:** salvage radical prostatectomy, prostate cancer, salvage therapy, robotic prostatectomy, recurrence

## Abstract

Prostate cancer is the second most common cancer among men, with many treatment modalities available for patients, such as radical prostatectomy, external beam radiotherapy, brachytherapy, high-intensity focused ultrasound, cryotherapy, electroporation and other whole-gland or focal ablative novel techniques. Unfortunately, up to 60% of men with prostate cancer experience recurrence at 5 to 10 years. Salvage radical prostatectomy can be offered as an option in the setting of recurrence after a primary non-surgical treatment. However, the complexity of salvage radical prostatectomy is considered to be greater than that of primary surgery, making it the least popular treatment of choice. With the wide use of robotic platforms in urologic oncologic surgery, salvage radical prostatectomy has attracted attention again because, compared to past data, modern series involving salvage Robot-Assisted Radical Prostatectomy have shown promising results. In this narrative literature review, we comprehensively examined data on salvage radical prostatectomy. We investigated the correlation between the different types of primary prostate cancer therapy and the following salvage radical prostatectomy. Furthermore, we explored the concept of a robotic approach and its beneficial effect in salvage surgery. Lastly, we emphasized several promising avenues for future research in this field.

## 1. Introduction

Prostate cancer (PCa) is the second most common cancer among men (after skin cancer), with an estimated 1.4 million diagnoses worldwide in 2020 [1]. About one-third of individuals with locally advanced prostate cancer (PCa) receive non-surgical first-line therapy with curative aims [2], including external beam radiotherapy (EBRT), brachytherapy, high-intensity focused ultrasound (HIFU), cryotherapy, electroporation and other whole-gland or focal ablative novel techniques. Still, while these treatment modalities offer excellent and comparable long-term survival, up to 60% of these patients experience PCa recurrence at 5 to 10 years [3,4,5]. In the majority of patients, the recurrence is limited to the prostate and is potentially curable [6]. Salvage radical prostatectomy (sRP) is a therapeutic option for PCa recurring after a primary non-surgical treatment. Although sRP offers the chance of a cure, up to 90% of men undergo long-term palliative androgen deprivation therapy (ADT) [2], perhaps owing to the complexity of salvage therapy, thus experiencing ADT-related comorbidities, such as diabetes mellitus, cardiovascular and peripheral vascular disease, depression and venous thromboembolism [7,8], and also remain uncured. This trend is mostly based on past sRP data, which demonstrated significant complication rates and poor continence results. These are thought to be due to the radiation-induced development of fibrosis and adhesions, which usually [9] alter the surgical planes and anatomic landmarks, making sRP a challenging task, even in expert hands [10]. However, sRP has attracted attention again recently because, compared to past data, modern series involving salvage Robot-Assisted Radical Prostatectomy (sRARP) have shown promising results [11]. Better vision, enhanced dexterity and superior tissue manipulation are advantages of robotic techniques that are thought to be of paramount importance in surgery of such high complexity. It is also worthy of mention that the surgical outcomes can be significantly affected by the type of primary prostate cancer therapy, and therefore the surgical technique should be adapted accordingly. Hence, it is essential to carefully select patients for sRP in order to avoid exposing men who will not benefit from the operation to surgery-related risks.

Taking the above into consideration, we tried to provide an up-to-date comprehensive literature review by consolidating the data regarding the current role of sRP in the treatment of patients facing PCa recurrence.

### 1.1. Patient Selection Criteria for sRP

Generally, candidates for sRP are those diagnosed with localized PCa following biochemical recurrence (BCR) after primary EBRT or focal therapy (FT) with a post-treatment biopsy confirming recurrent PCa. According to the European Association of Urology (EAU) guidelines, sRP should be considered only in patients with low co-morbidity, a life expectancy of at least 10 years, pre-sRP PSA levels < 10 ng/mL and an initial biopsy ISUP grade ≤ 2/3, no lymph node (LN) involvement or evidence of distant metastatic disease pre-sRP and those whose initial clinical staging was T1 or T2 (Table 1) [5,9]. BCR after EBRT, with or without short-term hormonal manipulation, is defined (with an accuracy of > 80% for clinical failure) by the American Society for Therapeutic Radiology and Oncology (ASTRO) as any PSA increase > 2 ng/mL higher than the PSA nadir value, regardless of the serum concentration of the nadir [12]. After focal therapies such as HIFU or cryotherapy, no endpoints have been validated against clinical progression or survival; therefore, no consensus has been reached on a definition for BCR following these alternative local treatments [13,14]. Regarding the role of imaging in the assessment of recurrence prior to a salvage treatment, MRI has shown outstanding results in diagnosing local recurrence [15,16,17,18] and can be utilized for biopsy targeting and guiding local salvage treatment, even though it somewhat underestimates the extent of the local recurrence [19]. Considering the high morbidity of salvage therapies following ΕΒRT, distant metastases must be ruled out before proceeding to these treatments. Bone scan as well as computed tomography (CT) have mostly been used in the past in order to identify bone or nodal metastases. Prostate-specific membrane antigen positron emission tomography CT (PSMA-PET/CT) has emerged as a new imaging modality that might replace previous ones, as it demonstrates the highest sensitivity in detecting PCa recurrence [20,21].

### 1.2. sRP vs. sRARP

Currently, minimally invasive radical prostatectomy is the gold-standard procedure for the surgical treatment of localized PCa. The evolution of radical prostatectomy towards robotic surgery was also adopted in the salvage setting after demonstrating better results than the open approach. Kenney et al., after retrospectively comparing sRP with sRARP, demonstrated that the estimated blood loss (EBL) was significantly lower in the robotic group versus the open group (381.3 mL versus 865.0 mL, *p* = 0.001), however, without any notable differences in the rate of intraoperative complications, postoperative complications or oncologic outcomes [22]. In a larger series comparing sRP with sRARP, patients treated with the robotic approach experienced less anastomotic strictures and blood loss and improved continence outcomes with no statistical difference in BCR [23]. The aforementioned results are line with a study from Gontero et al. which collected a significant amount of data from eight tertiary referral centers between 2000 and 2016, including 395 salvage radical prostatectomies, of which 186 were open and 209 were robotic [11]. Robotic salvage radical prostatectomy yielded lower blood loss and a shorter hospital stay (each *p* < 0.0001), and anastomotic strictures were more frequent with open salvage radical prostatectomy (16.57% vs. 7.66%, *p* < 0.01) [11].

As rectal injuries are of major concern in salvage procedures, many surgeons have adopted the Retzius-sparing approach in the salvage setting, as it provides direct view of the rectum, allowing for meticulous tissue handling and avoiding related complications. It also provides better functional outcomes regarding urinary continence. Madi et al., in one of the first studies reporting the outcomes of salvage Retzius-sparing RARP (sRS-RARP), presented that the patients who underwent sRS-RARP had better immediate (25.0% vs. 0.0%, *p* < 0.001), 3-month (80.0% vs. 0%, *p* < 0.001) and 12-month continence rates (100% vs. 44%, *p* = 0.0384) compared to patients that had a conventional sRARP [24]. Also, the rates of positive surgical margins (PSMs) and BCR were not significantly different between the two groups. When it came to complications, the 30-day complication rates were lower in the sRS-RARP group (%, 1/20) than the sRARP group (18%, 1/5) (*p* = 0.42), though they were not significantly different [24]. Similarly, another more recent comparative study of sRS-RARP and sRARP which included a larger number of patients concluded that sRS-RARP provides significantly improved continence preservation with no differences in its complication rates or oncologic outcomes [25]. At a median follow-up of 23 months, the sRS-RARP group had improved continence (78.4% vs. 43.8%, *p* < 0.001 for 0–1 pad, 54.1% vs. 6.3%, *p* < 0.001 for 0 pad), used a lower number of pads per day (0.57 vs. 2.03, *p* < 0.001) and had an earlier return to continence (median 47 vs. 180 days, *p* = 0.008) [25].

### 1.3. Surgical Steps and Technical Modifications

The surgical steps of both sRP and sRARP are very similar to those of standard RP, but some adaptations based on the primary treatment are usually made during some critical stages of the operation. The first step of the robotic procedure, the so-called bladder drop, must be carried out with caution, as neo-vascularization post radiotherapy can cause extensive bleeding. The endopelvic fascia dissection, much like with standard RP, is carried out near the base of the prostate to avoid bleeding and damage to the sphincter. Following hormonal treatment, the seminal vesicles shrink and usually become strongly attached to the surrounding tissues, making it difficult to take them en bloc with the prostate. Posterior dissection can also become challenging, as normal tissue is replaced with fibrotic tissue and adhesions. In this case, blunt dissection and minimal or no use of thermal cautery are crucial to avoid rectal injury [26].

Apex dissection is another crucial step that requires careful manipulation. Post brachytherapy, radioactive pellets may be present at the location of the apex, and excessive traction could lead to fracture of the prostate. Some surgeons have suggested omitting ligation of the dorsal vein and completely freeing the lateral margins of the prostate to allow for full mobilization of the prostate [27]. This was based on the observation that dorsal venous complex bleeding is minimal even without ligation in the salvage setting due to radiation-induced changes [28]. When the primary treatment modality is cryotherapy, the apex is also fixed to the area, and the treated areas appear as cavities with fibrosis. In this case, dissection proceeds from the midline to the lateral side with extra caution at the apex [29]. By contrast, in patients previously treated with HIFU, the region being treated is a cavity devoid of fibrosis, meaning careful dissection is necessary to avoid viable tumors entering these cavities. The dissection occurs in the extra-fascial plane from the lateral to the medial treated side, and the rectum is dissected last. These steps are followed because in HIFU, the ultrasound energy is transferred into the prostate by means of a transrectal probe, resulting in the prostate becoming immobilized along the midline. Since bladder neck contracture is a common complication after sRP, with a five-fold higher incidence than after primary RP [30], some technical modifications have to be made. To reduce the incidence of bladder neck contracture, extensive excision of the bladder neck and reconstruction are advised in cases of severe fibrosis. However, this has to be performed carefully in order to avoid urinary leakage. Anastomotic leaks represent a frequent complication with relatively high rates [31]; thus, performing a cystogram before catheter removal is recommended. A tennis racquet reconstruction or a Rocco stitch to approximate the tissues might also prevent a possible leakage. Aiming to reduce the incidence of incontinence after surgery, Ogaya-Pinies et al. demonstrated the innovative use of scaffolding tissue biografts, which can be sutured to Denonvilliers’ fascia to reinforce the posterior aspect of the vesicourethral anastomosis [32]. This scaffold was employed in addition to a posterior reconstruction of Denonvilliers’ fascia. The investigators demonstrated lower rates of anastomotic leaks (6.7% versus 35.5%) and a reduction in catheterization time of approximately 6 days compared to the control group.

As far as nerve sparing in sRP is concerned, this is an extremely contentious issue. Critics contend that we should not compromise the oncologic safety of our patients [28], which becomes more pronounced with recurrent disease, while proponents believe that this is a feasible option in well-selected patients [33,34]. One potential strategy entails the preservation of the contralateral nerve bundle subsequent to primary focal therapy, provided that magnetic resonance imaging (MRI) reveals no evidence of involvement. Regardless of whether nerve sparing is performed, bilateral pelvic lymphadenectomy is essential for staging and management of the disease given that lymph nodes yield a high positive rate after salvage surgery [11,33]. Taking into consideration that the pelvic lymph nodes are typically excluded from normal radiation templates, lymphadenectomy for sRARP is typically not a more technically challenging procedure than it is in the primary setting.

Also worthy of mention are the surgical techniques applied during the management of rectal injury, which is considered a nightmare for most surgeons. Despite presenting a decrease in recent years due to the magnified vision that robotic surgery offers, it is still the most feared complication. When rectal injury is detected, repair of the rectal defect is not advised. Since prior prostatic radiation is a significant risk factor for rectourinary fistula formation [35], a temporary diverting colostomy has to be the first-line treatment option [36].

### 1.4. Salvage Radical Prostatectomy after Radiotherapy—Oncologic and Functional Outcomes

Data for sRP following radiotherapy are mostly retrospective and, to a lesser extent, prospective from multi- and single-institution studies, while there are no data from randomized controlled trials (RCTs) (Table 2). Multi-institutional retrospective studies present the largest populations. In one early study on sRP after radiotherapy, Ward et al. reviewed and analyzed data from 199 patients who underwent open salvage surgery after radiotherapy at their center, presenting a median progression-free survival (PFS) of 8.7 years and a 10-year cancer-specific survival (CSS) rate of 77% [37]. In this study, several pathological features of the removed prostate were evaluated as predictive factors, with ploidy demonstrating significant predictive power for PFS (*p* < 0.05). In the following years, more centers presented their data, including on patients treated with sRARP [38,39,40]. Improvements in surgeons’ experience and the use of robots have underlined that sRP and sRARP after radiotherapy can achieve excellent tumor control with favorable functional outcomes. Most centers present excellent CSS rates, which range from 88.7% to 98% at 5 years and reach 83% at 10 years after sRP [41,42,43], and promising 5-year BCR-free rates [44]. By contrast, Mohler et al. published the results of CALGB 9687 (Alliance), a prospective multi-institutional salvage prostatectomy series, demonstrating a relatively low 10-year BCR-free rate of 33% and a 10-year OS rate of 52% [45]. While these low rates might reflect the poor quality of the design of former studies, which were mostly retrospective, the PSM rate of 17% presented by the authors is in line with the published literature, highlighting the advancements in surgical techniques [41,46]. Except for improvements in surgical technique, the wise selection of patients plays a key role in the oncologic outcomes. In an attempt to validate the previously mentioned EAU patient selection criteria for sRP, Calleris et al. retrospectively reviewed 1265 patients who underwent sRP at 14 referral centers (2000–2021), stratified by compliance with them [5]. They showed that the EAU-compliant group experienced more favorable pathological and functional outcomes (79% vs. 63% wearing no pads at 1 year; *p* < 0.001) and had a significantly better metastasis-free survival (MFS) (90% vs. 76% at 5 years; *p* < 0.001), prostate-specific-antigen-free survival (55% vs. 38% at 5 years; *p* < 0.001) and overall survival (89% vs. 84% at 5 years; *p* = 0.01) [5]. Men who did not meet the criteria had a higher risk of metastasis, and their benefit from surgery might have been significantly less than that for patients who did meet the EAU criteria. Taking into account these results and the perioperative risks associated with surgical intervention, it becomes obvious that identifying patients who will benefit from sRP is of paramount importance.

When contemplating the functional outcomes of post-radiation sRP, it is noteworthy that erectile dysfunction and incontinence persist as prevalent limitations of the aforementioned procedure. Besides oncological efficacy, which is the most critical endpoint, urinary incontinence is a significant and long-term consequence that substantially decreases patients’ quality of life. While the definition of continence may vary between centers (most commonly referred to as 0–1 pad/day), continence rates seem to have increased slightly over time, probably due to ever-growing surgical experience and the wider adoption of robotic surgery. In a study from Onol et al., continence rates presented a considerable discrepancy when the authors used two definitions of continence, underscoring the need for agreement on the terminology used [44]. The overall full (no pads) and social (0–1 pad/day) continence rates at 1 year were 87.5% vs. 51.3% (*p* = 0.002) [44]. Eandi et al., in one of the first studies assessing continence after sRARP, presented a high incontinence rate of 67% [47]. However, some studies published in the following years demonstrated a gradual increase in continence rates [11,45]. Despite the surgeon’s experience and expertise, which, as already mentioned, affect the continence outcomes, Calleris et al. showed that organ-confined prostate cancer ≤ stage T2b, a pre-RP Gleason score ≤ 7 and pre-sRP PSA levels < 10 ng/mL are associated with better post-sRP continence [5].

Given that erectile function is typically low in most patients before sRP, discerning and evaluating changes specific to the surgery appear to be a difficult task. In a propensity-matched comparative study of 135 patients, Nathan et al. found that compared to patients who underwent sRARP, those who underwent primary RARP demonstrated better potency results (94.8% sRARP vs. 76.3% pRARP, *p* < 0.001) [48]. Despite this, one can easily recognize a clear trend of high erectile dysfunction following salvage surgery treatments, with the erectile dysfunction rates at 1 year ranging from 45% to 100% [11,45,47,49,50]. We also have to mention that, similar to the definition of incontinence, the outcome measurements and definitions for erectile dysfunction after sRP varied between different studies (with different questionnaires used).

**Table 2:** Studies of considerable interest assessing oncologic and functional outcomes for salvage radical prostatectomy after radiotherapy.

Study	Number of Patients	Oncologic Outcomes	Functional Outcomes
Ward et al. (2005) [37], retrospective, single-center	199	BFS = 48%, CSS = 77%	Incontinence at 1 Year = 33% Erectile Dysfunction at 1 Year = N/A
Chade et al. (2011) [41], retrospective, multicenter	404	BFS = 37% MFS = 77% CSS = 83% OS = 77%	N/A
Mandel et al. (2016) [42] Retrospective, single-center	55	BFS = 73.9%	Incontinence at 1 Year = 26%
Pisters et al. (2009) [43] Retrospective, single-center	42	BFS = 61% OS = 95%	N/A
Onol et al. (2020) [44] Retrospective, single-center	126	BFS = 56%	Incontinence at 1 Year = 49.7% Erectile Dysfunction at 1 Year = 87%
Mohler et al. (2019) [45] Prospective, multicenter	41	BFS = 33% OS = 52%	Incontinence at 1 Year = 40% Erectile Dysfunction at 1 Year = 45%
Gontero et al. (2019) [11] Retrospective, multicenter	395	N/A	Incontinence at 1 Year = 43% Erectile Dysfunction at 1 Year = 85%
Eandi et al. (2010) [47] Retrospective, single-center	18	N/A	Incontinence at 1 Year = 67% Erectile Dysfunction at 1 Year = 100%
Catarino et al. (2022) [49] Retrospective, single-center	29	BFS = 50%	Incontinence at 1 Year = 79% Erectile Dysfunction at 1 Year = 85%
Stephenson et al. (2004) [50] Retrospective, single-center	100	N/A	Incontinence at 1 Year = 61% Erectile Dysfunction at 1 Year = 84%

BFS = biochemical recurrence-free survival, CSS = cancer-specific survival, MFS = metastasis-free survival. OS = overall survival, N/A = not available.

### 1.5. Salvage Radical Prostatectomy after Focal Therapy

Focal therapy (FT) is a treatment strategy that is being used more and more for localized prostate cancer. It utilizes different energy modalities to precisely target the tumor and minimize the effects of the treatment on surrounding healthy prostate tissue and structures. In recent years, FT has become a viable therapeutic option thanks to advances in imaging, which have improved our ability to locate the cancer within the gland. The primary objective of FT is to selectively treat specific areas of the prostate gland that have clinically significant cancer. Consequently, it has a reduced likelihood of causing erectile dysfunction, urine incontinence and gastrointestinal issues [51].

Despite their inferior oncological results, the main attraction of focal therapies is the preservation of erectile and urinary function. The energy sources commonly used for FT include HIFU, focal laser ablation (FLA), focal cryotherapy, irreversible electroporation (IRE) and photodynamic therapy (PDT). The core of focal therapy is that the index lesion or dominant tumor remains the primary origin of metastasis [52,53]. There is a lack of long-term follow-up data, re-treatment is often required and salvage treatment is complicated due to periprostatic scarring and fibrosis. In conclusion, focal therapy has the goal of destroying the index lesion, but men enlisting for focal ablation should consider that the likelihood of residual cancer foci is high, and the need for salvage treatment may be imminent.

Before the introduction of RARP, the option of open radical prostatectomy after the failure of localized therapies was not a comfortable choice.

HIFU is the most popular noninvasive modality and uses ultrasound waves delivered transrectally to ablate prostate tissue through coagulative necrosis by heating these tissues over 65 °C. The failure rate of partial gland ablation ranges between 35% and 42%, with approximately 14% in-field recurrence. The ED rates range from 0% to 25%, and other rare complications include incontinence and rectourethral fistula [54,55].

Cryotherapy is based on the principle of subjecting tissues to temperatures below minus 30 degrees Celsius, resulting in cellular demise. During cryotherapy, metal probes are inserted transperineally into the prostate under transrectal ultrasound guidance. The probes are loaded with argon gas that induces freezing of the adjacent prostate tissue and, in turn, results in cell death by necrosis and apoptosis [56].

Photodynamic therapy (PDT) involves the intravenous administration of photosensitizers, which, when activated by the light delivered by optical fibers inserted transperineally into the prostate, produce cytotoxic reactive oxygen species. The generation of reactive oxygen species causes increased hypoxia, which leads to vascular damage and cancer cell necrosis. Azzouzi et al. published a multicenter randomized controlled trial comparing PDT to AS in patients with low-risk PCa [57]. Interestingly, the PCa progressed in 28% (*n* = 206) of the patients in the PDT group, as compared to 58% (*n* = 207) of the patients in the AS group. However, the erectile dysfunction rates and the urinary complications were higher in the PDT group, at 38% versus 11%, respectively [58]. These results should be approached with caution, as the patients did not undergo mpMRI and confirmatory or saturation biopsy prior to selection into the treatment groups. This limitation could also explain the high rates of progression reported in the active surveillance group (nearly 60%) [59].

FLA, another focal therapy, works in a similar way to the treatment modalities already discussed. In this procedure, a laser fiber is carefully inserted into the cancerous tissues, either through the transperineal or transrectal route. The energy transmitted through the fiber causes the targeted cells to undergo necrosis. FLA is a viable and safe option for treating localized PCa [60]; however, most of the data come from studies with small sample sizes and short follow-ups. Lepor et al. published their results on 25 consecutive patients with low–intermediate-risk PCa treated with MRI-guided FLA [61]. Post ablation biopsy at 3 months showed no evidence of cancer in 96% of the patients without a compromise in the functional outcomes [59].

IRE includes the placement of electro-needle probes through the perineum into the ablative target under ultrasound or MRI guidance. The goal of IRE is permeabilization of the cell membrane by employing the high-voltage electrical pulses of two or more electrodes placed in the prostate, resulting in a loss of homeostasis and consequential cell death. Van den Bos et al. investigated 63 patients with low- and intermediate-risk PCa treated with IRE [62] and reported a 16% in-field recurrence rate. The urine symptom score did not change 6 months after the surgery, and there was a slight decrease in the sexual quality of life score from 66 to 54 [59]. After IRE, fibrosis at the ablated site with adherence to the pelvic floor, the neurovascular bundle or the posterior plane was often noted. The surgeons reported that primary dissection of untreated tissue (e.g., prostatic pedicles, posterior plane, apex of the prostate and nerve sparing) improved the three-dimensional visualization of the prostate. In a retrospective subset analysis by Luigi A.M.J.G. van Riel et al., a total number of 39 patients underwent sRP after IRE, and no significant perioperative complications were observed. In addition, after a median follow-up of 17.7 months, urinary continence and erectile function were preserved in 34 (94.4%) and 18 patients (52.9%), respectively, while their quality of life remained stable [63].

T. Spitznagel et al. showed that operative time, length of stay, nerve sparing and blood loss are not statistically different between sRARP after HIFU and primary RARP. On the other hand, 30 d postoperative complications are more common in sRARP (46.2% vs. 26.9%), and more specifically, the Clavien–Dindo complication rate was 30.8% in an sRARP group and 7.7% (2/26) in a pRARP group [64]. Nunes-Silva et al. reported no statistically significant differences in their matched-pair comparison (*p* = 0.74), but the rates of bilateral nerve sparing were significantly lower with sRARP (*p* = 0.016), perhaps suggesting that nerve bundle tissues presented some level of impairment that compromised adequate preservation (Table 3) [65]. Last but not least, in a recent meta-analysis, Fernando Blank et al. showed acceptable complication rates of 12.2% of patients experiencing some sort of postoperative complication, of whom 18 (4.6%) met the criteria for a major complication (CG ≥ 3), ranging from 0 to 31% across all studies [66]. In a matched analysis, K.R. Seetharam Bhat et al. analyzed retrospectively the outcomes of sRARP after focal ablation for PCa in comparison to primary RARP. Primary RARP was performed faster (median difference of 11 min; 95% CI 3–19; *p* = 0.007), the rate of bilateral nerve sparing was 32% higher, and while there were no significant differences in the postoperative Clavien–Dindo scores, the incidence of lymphoceles was 15% lower than with salvage RARP after focal therapy [67].

In a comparative effectiveness study, Arjun Nathan et al. collected data on 135 patients and compared the outcomes of salvage RARP after whole-gland (such as radiotherapy, brachytherapy and whole-gland HIFU) and focal gland therapies (such as focal HIFU, cryotherapy and electroporation). There were no statistically significant changes in operative time, estimated blood loss, transfusion or duration of stay between whole- and localized gland therapy. Unilateral and bilateral nerve-sparing procedures were possible in 8.2% and 2% of the entire-gland group, respectively, compared to 31.4% and 4.7% of the focal group. This difference was attributed to technical feasibility and the location of the tumor (*p* = 0.001) [68].

The main concern with a salvage radical prostatectomy is the increased risk of rectal injury. A recent meta-analysis showed that intraoperative complications were rare, and there was not any rectal injury, despite what focal therapy patients underwent. Also, postoperative complications are not that common, and only 12.2% of patients experienced one, most of which were UTIs, anastomotic leaks and wound infections [66].

Patients who undergo focal therapy choose quality of life over the better oncological outcome of radical therapies. So, high rates of post-surgical incontinence and erectile dysfunction play a detrimental role in accepting the choice of salvage radical prostatectomy. A retrospective and multicenter cohort study by Lorenzo Marconi et al. showed that the continence rate at 12 months was 83% [69]. As far as potency is concerned, the preoperative erectile dysfunction rate in this study cohort was 33%, and nerve-sparing surgery was performed in 76% of the patients (bilateral = 33%, unilateral = 37%, incremental bilateral = 4% and incremental unilateral = 2%) [69]. The absolute potency rate at 12 months after surgery was 14%. Despite the postoperative continence rates, the perioperative outcomes, positive margin rates and complications observed are comparable to those previously reported for series of patients undergoing primary RARP [70,71], while sRARP confers worse erectile function results compared to RALP in treatment-naïve patients [72].

**Table 3:** Studies of considerable interest assessing oncologic and functional outcomes for salvage radical prostatectomy after focal therapy.

Study	Number of Patients	Oncologic Outcomes	Functional Outcomes
Marconi et al. (2019) [69] Retrospective, multicenter	82	BCR = 21%	continence rate = 83% erectile function rate = 14%
Onol et al. (2020) [44] Retrospective, single-center	32	BCR = 19%	continence rate = 78% erectile function rate = 28%
Nunes-Silva et al. (2017) [65] Retrospective, single-center	22	BCR = 32%	continence rate = 32% erectile function rate = 0%
Spitznagel et al. (2021) [64] Retrospective, single-center	13	BCR = 0%	continence rate = 23% erectile function rate = 89%
De Groote et al. (2020) [29] Retrospective, single-center	61	BCR = 18%	continence rate = 39% erectile function rate = 5%

BCR = biochemical recurrence.

## 2. Application of Artificial Intelligence in Salvage Radical Prostatectomy

In recent years, there has been a growing trend in the use of artificial intelligence (AI) in robotic surgery and especially in oncologic procedures such as radical prostatectomy, which affect a great many people. In a recent review, Bellos et al. summarized the different applications of AI and machine learning in oncologic surgical procedures, demonstrating their wide application in urology and more specifically in RARP [73]. For example, live assistance of the surgeon during RARP with the use of AI was introduced by Checcucci et al., who presented a highly advanced artificial neural network model for predicting intraoperative bleeding [74]. In another application of AI to intraoperative assessment, researchers used machine learning models to analyze color and texture in order to identify the anatomical structures during surgery [75]. Even though most applications of AI are utilized in RARP, an extrapolation of their results to the setting of salvage procedures could potentially help surgeons improve their outcomes and, in turn, benefit their patients.

## 3. Conclusions

The rapid improvement of robotic surgical platforms in recent years and the increased surgical experience of urologists have renewed interest in salvage radical prostatectomy. When performed by experienced surgeons, it is a safe procedure showing potential for further improved functional outcomes considering the ongoing development of robots and the use of AI to assist urologists in their daily practice.

## Figures and Tables

**Table 1:** European Association of Urology criteria for patient selection for salvage radical prostatectomy.

Low comorbidity Life expectancy of at least 10 years Pre-sRP prostate-specific antigen levels < 10 ng/mL Pre-sRP biopsy International Society of Urological Pathology grade group ≤ 2/3 No lymph node involvement or evidence of distant metastatic disease pre-sRP Initial clinical stage T1 or T2

sRP = salvage radical prostatectomy.
